# Supplementary material for: LTBP3 Frameshift Variant in British Shorthair Cats with Complex Skeletal Dysplasia
Source: Genes (Basel). 2021 Nov 29;12(12):1923. doi: 10.3390/genes12121923 (PMC8701722; doi:10.3390/genes12121923)
Supplement: Supplementary file 1 [file genes-12-01923-s001.zip › Supplementary_Figures_S1_S8.pdf]

Supplementary Figures

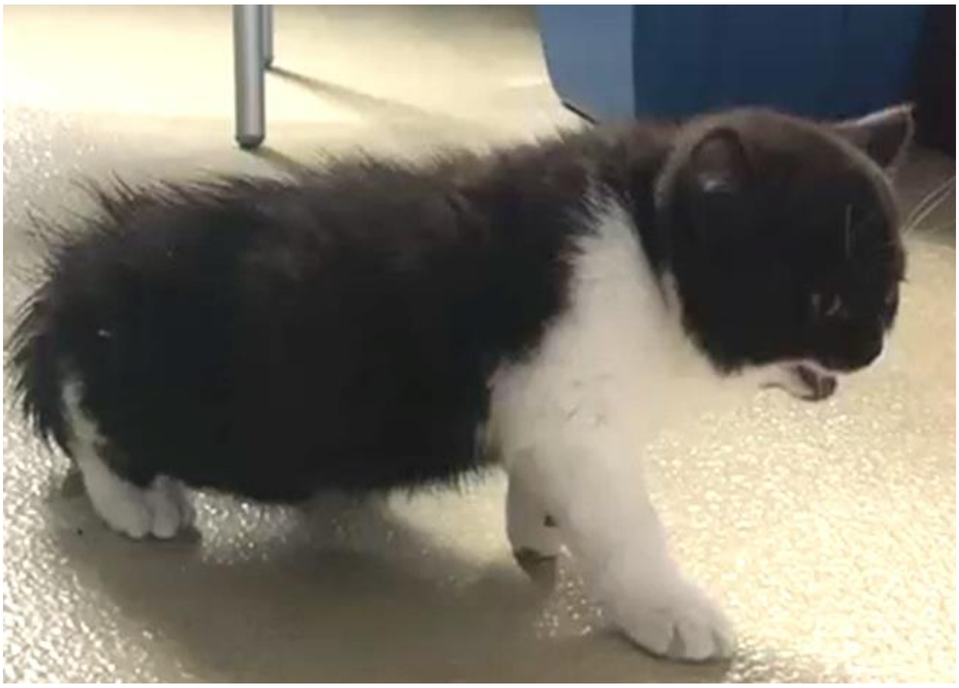

Figure S1. Photograph of the male affected kitten. Note the ambulatory paraparesis and the lordosis.

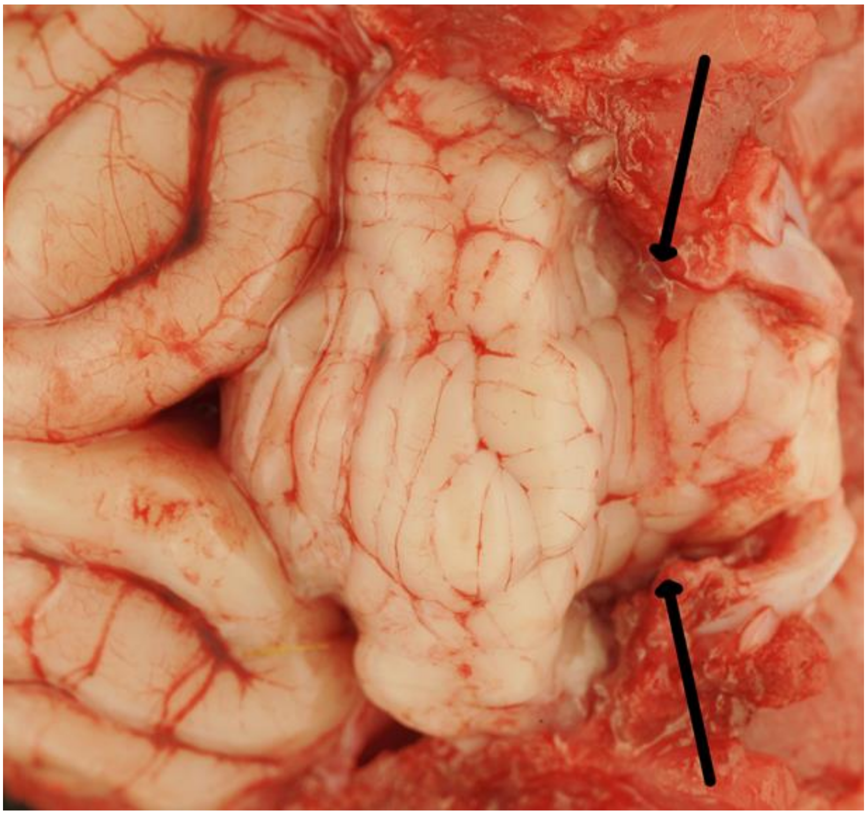

Figure S2. Male affected kitten, dorso-caudal cerebrum and cerebellum. The cerebellum shows dorsal compression (arrows) and herniation of the cerebellar vermis.

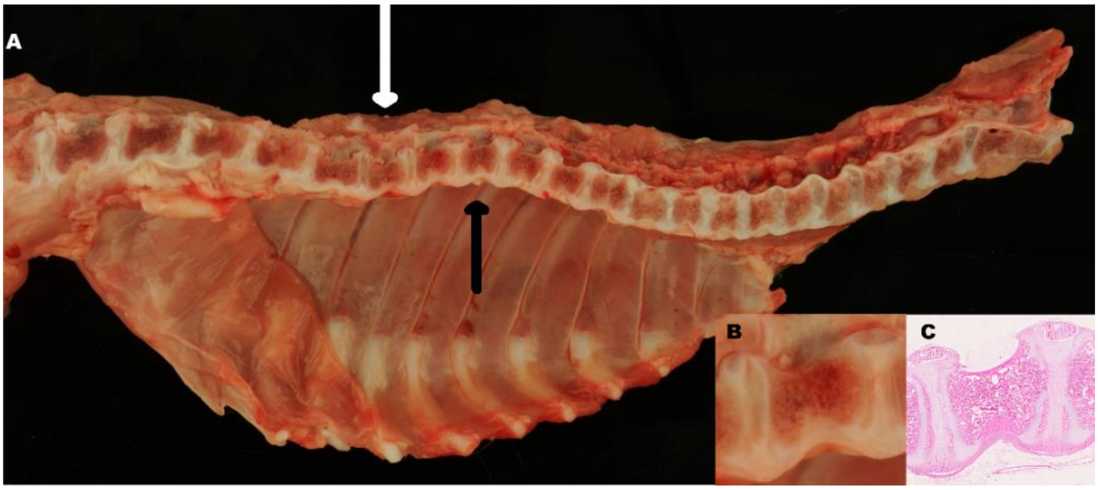

Figure S3. Male affected kitten, longitudinal cut of vertebral column with ribs. (**A**) Kyphosis (black arrow) and lordosis (white arrow) of thoracic vertebral column. (**B**) Higher magnification of Th9 with hourglass shaped vertebral body. (**C**) Corresponding HE stain of Th9 with depiction of the bone structure.

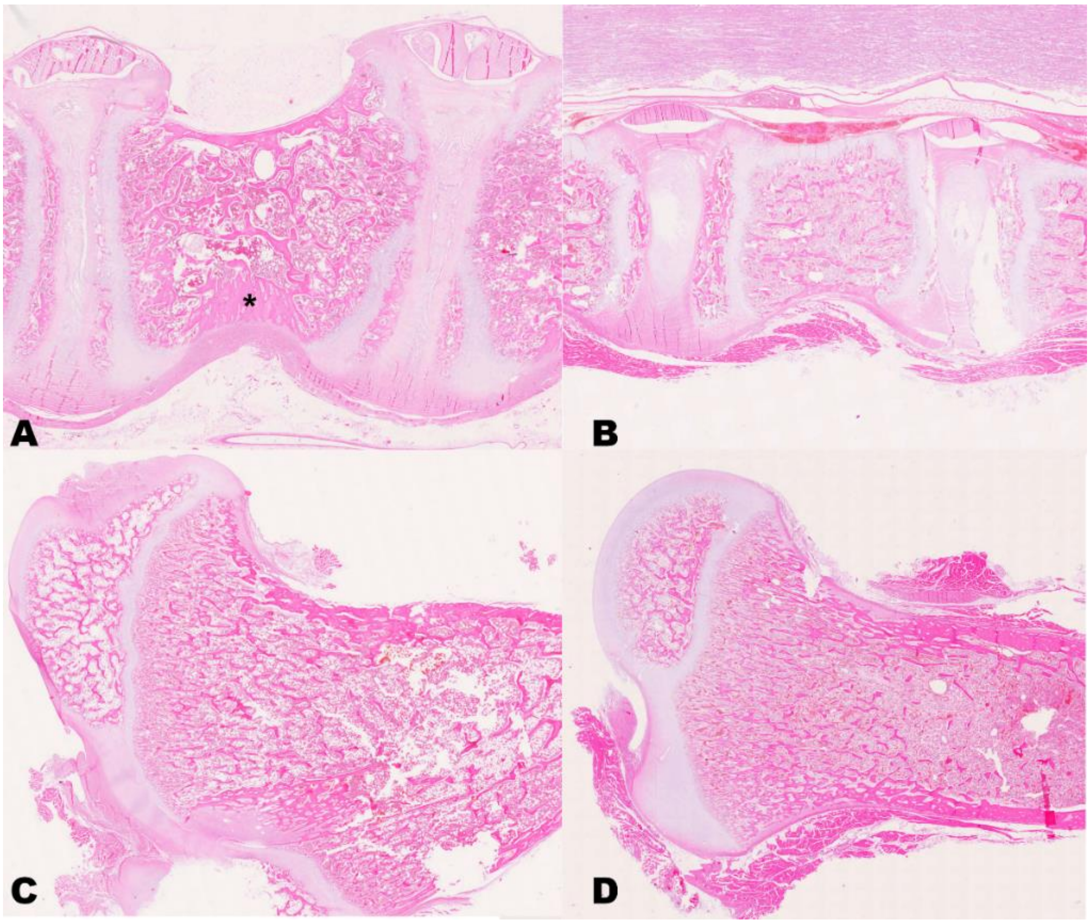

Figure S4. Longitudinal cut of thoracic vertebral column of the (**A**) male affected kitten and (**B**) an unaffected, age-matched kitten. Femur head of the (**C**) male affected kitten and (**D**) unaffected control. (**A**) Affected kitten with hourglass shaped vertebral bodies. The marginal ridges with growth plates and intervertebral discs show a prominent dorsal and ventral expansion. The ventral cortical laminar bone shows an increased density and thickening up to 1 mm (*). The woven bone of the vertebral body shows an irregular arrangement. (**B**) Thoracic vertebral column of an age-matched control European shorthair kitten with normal vertebral bodies. The cortical laminar bone is thin with sectional incomplete bone formation and preserved cartilage. The woven bone trabeculae show an overall longitudinal orientation with occasional cross bridging. Magnification 1.03x, HE. (**C**) The femoral head of the affected kitten shows an irregular shape of the epiphyseal bone with undulation of the epiphyseal plate cartilage. The trabecular bone of the metaphysis shows a regular ossification. However, compared to the bone architecture of the age-matched control kitten, the trabeculae appear rather randomly directed with an equable density throughout the metaphysis. (**D**) The femur of the unaffected kitten shows a regular architecture of the femoral head with directional growth and higher density of trabecular bone in the vicinity of the cortical bone (remodeling). Magnification 0.59x, HE.

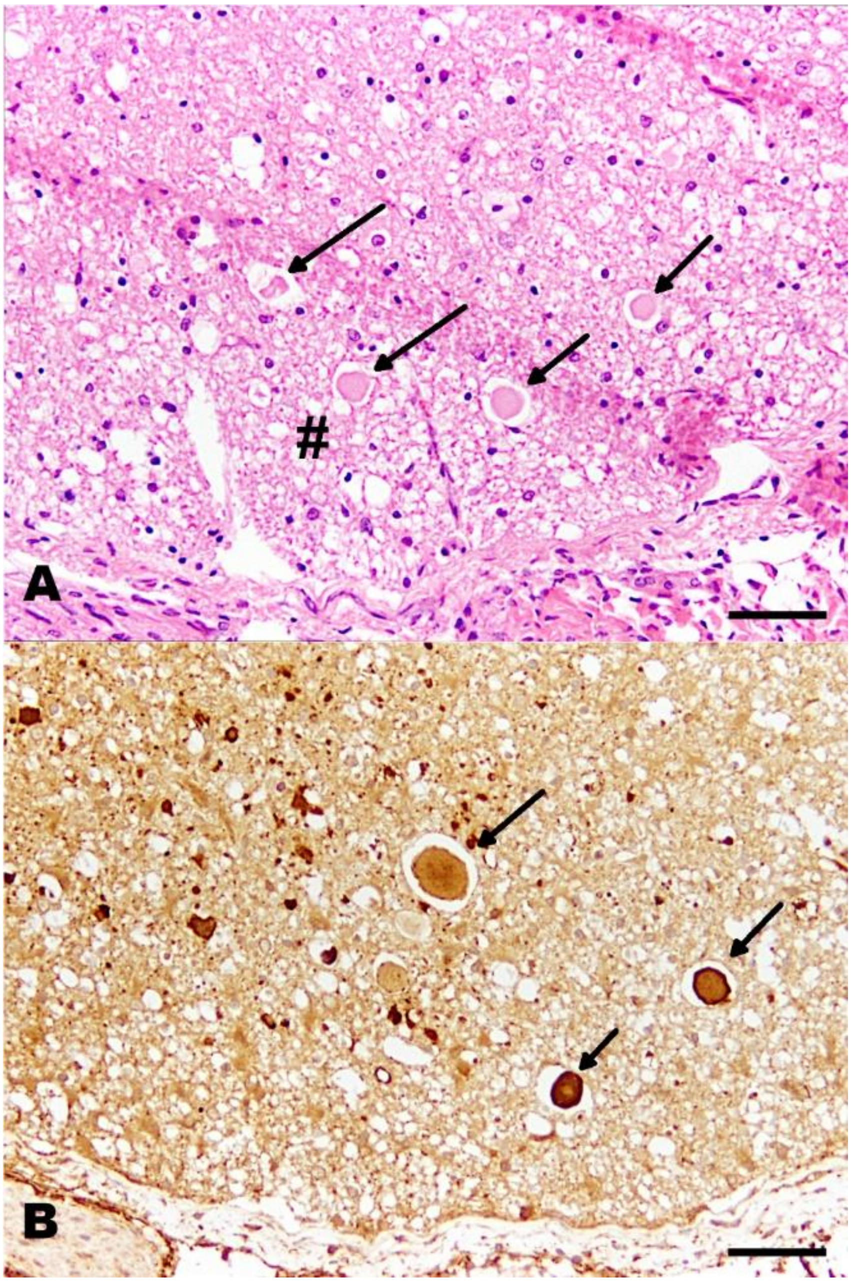

Figure S5. Male affected kitten, thoracic spinal cord, dorso-lateral funiculus. (**A**) loosening of myelin (#) with dilation of myelin sheaths and axonal swelling "spheroids" (arrows). Magnification 20x, bar 50 µm, HE. (**B**) Accumulation of beta-amyloid precursor protein (beta APP) in axoplasm (arrows). Magnification 20x, bar 50 µm, IHC, beta-APP.

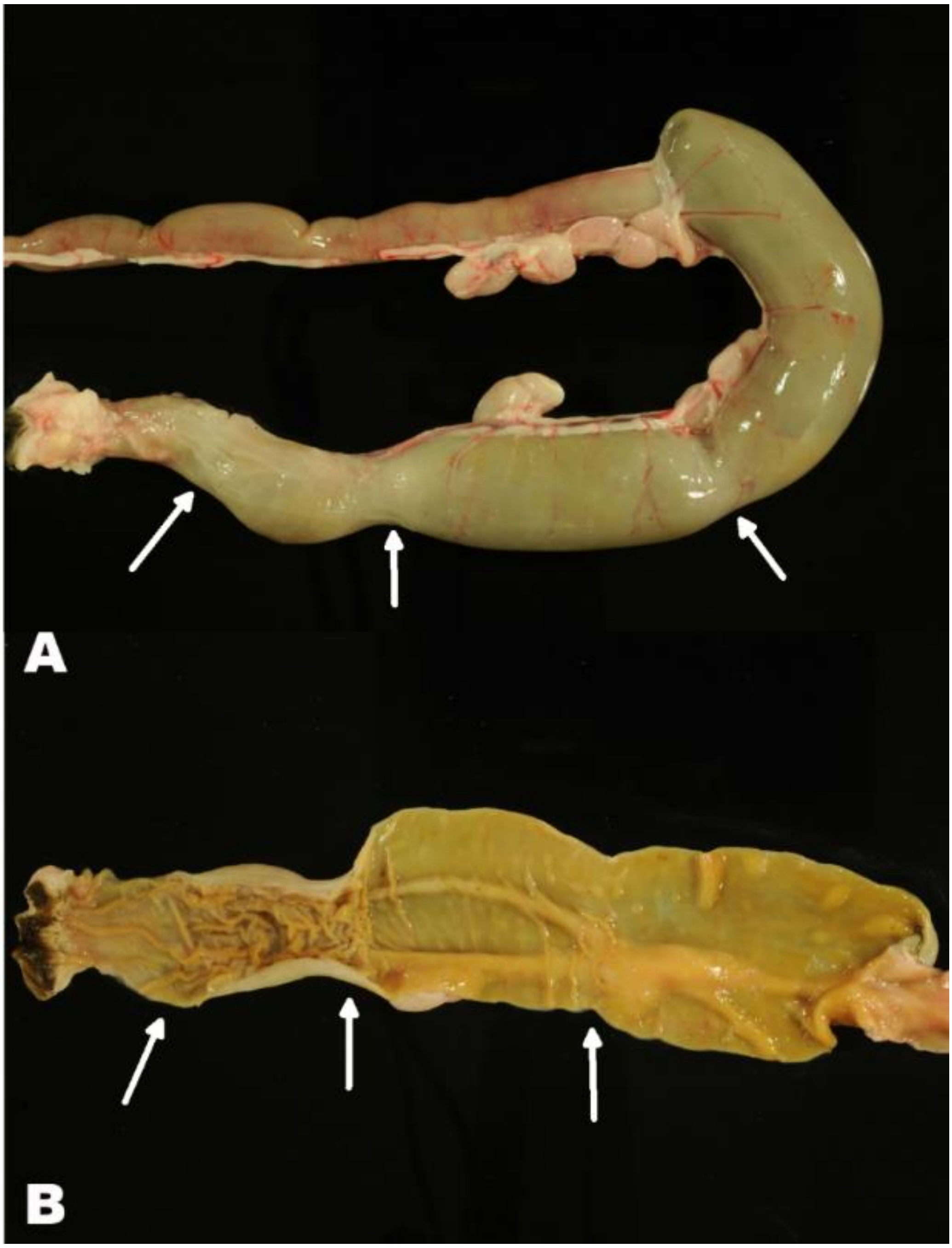

Figure S6. Male affected kitten, colon and rectum. (**A**) Dilation of colon with annular constrictions (white arrows) and distal contraction of rectum. (**B**) Colon and rectum opened up with increased thickness of the intestinal wall and wrinkling of the mucosa in the constricted areas (white arrows).

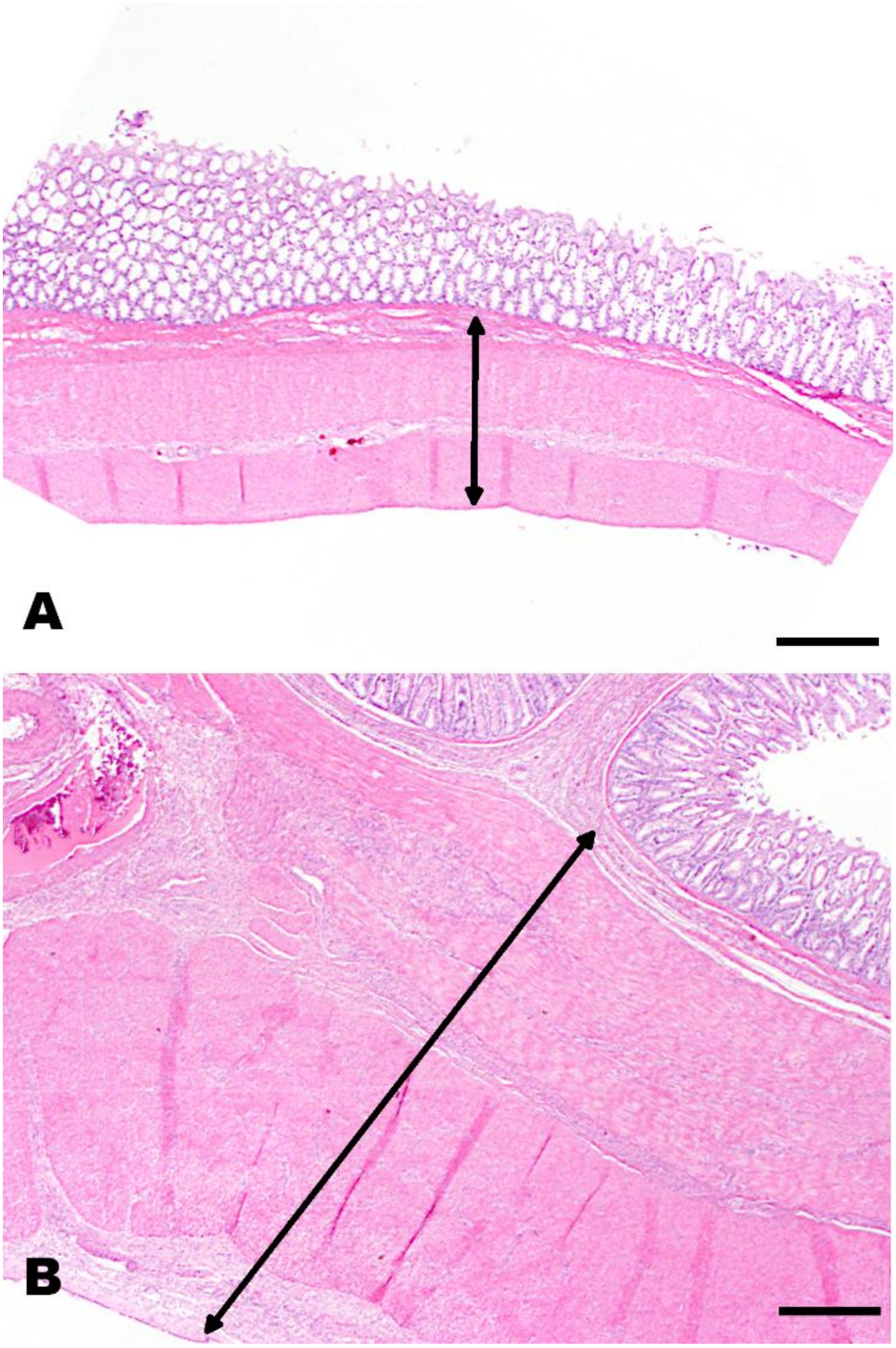

Figure S7. Male affected kitten, colon. (**A**) Dilated area of colon with normal thickness of intestinal muscle layers (double arrow: ~980 µm) and normal mucosa (~500 µm). (**B**) Constricted area of colon with marked hypertrophy and thickening of the muscle layers (double arrow: ~3290 µm) and inflammation with formation of granulation tissue between the muscle layers (see also Figure S8). The mucosa shows a normal thickness (~525 µm). Magnification 2x, bars 500 µm, HE.

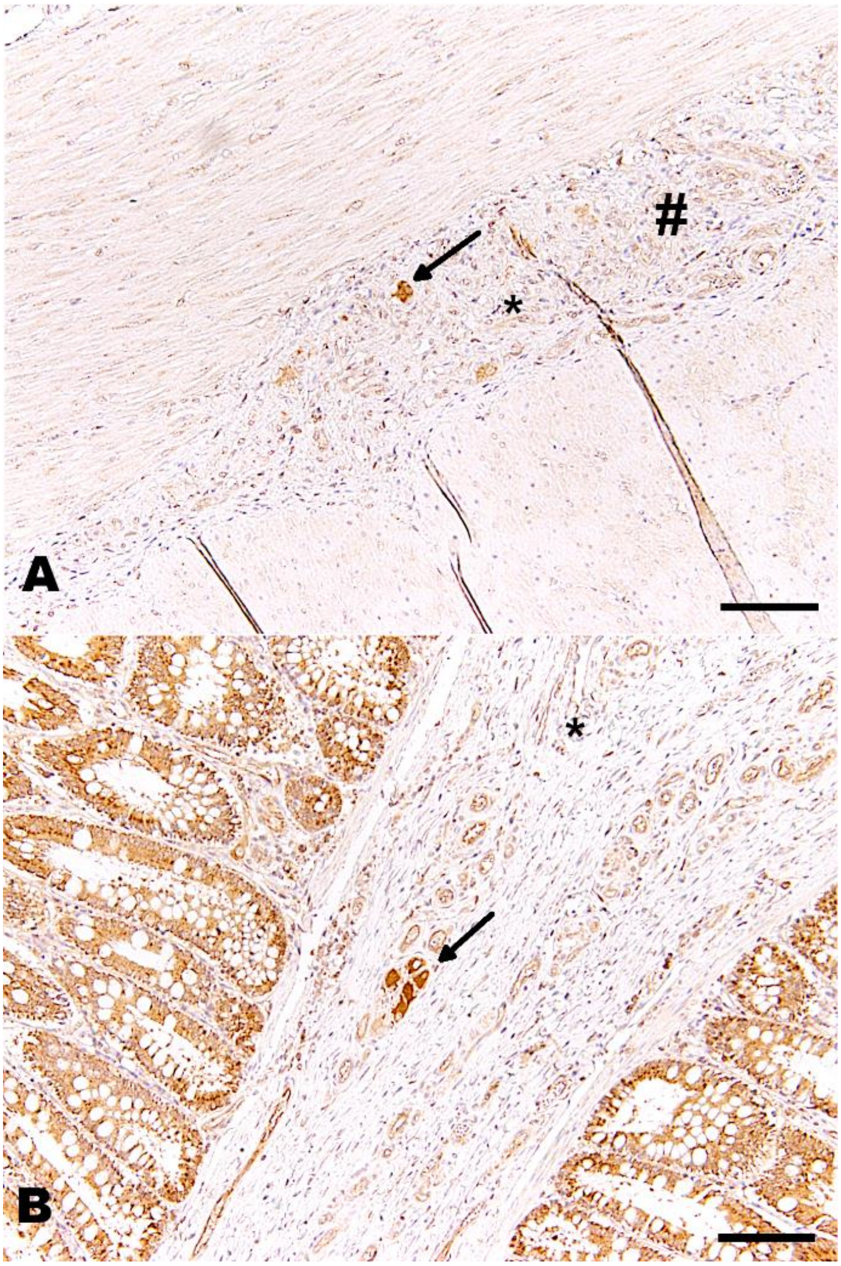

Figure S8. Male affected kitten, hypoganglionosis, colon. (**A**) Single neuron in the myenteric (Auerbach) plexus. (**B**) Four neurons in the submucosal (Meissner) plexus. Magnification 10x, bars 100 µm, IHC, n-NF. The thickening of the intestinal wall was accompanied by proliferation of vascularized fibrous connective tissue (granulation tissue) in the submucosa, between the muscle layers (*) as well as subserosal (*). Despite loss auf neurons, there was excessive proliferation of nerve fibers interwoven into the granulation tissue (#) and crossing the muscular layers.
